# Dynamic Graph Clustering Learning for Unsupervised Diabetic Retinopathy Classification

**DOI:** 10.3390/diagnostics13203251

**Published:** 2023-10-19

**Authors:** Chenglin Yu, Hailong Pei

**Affiliations:** 1 Key Laboratory of Autonomous Systems and Networked Control, Ministry of Education, Unmanned Aerial Vehicle Systems Engineering Technology Research Center of Guangdong, South China University of Technology, Guangzhou 510640, China; 2 Key Laboratory of Autonomous Systems and Networked Control, Ministry of Education, Unmanned Aerial Vehicle Systems Engineering Technology Research Center of Guangdong, School of Automation Science and Engineering, South China University of Technology, Guangzhou 510640, China; auhlpei@scut.edu.cn

**Keywords:** unsupervised diabetic retinopathy, dynamic graph clustering, topological relationship, consistency smoothing, deep learning

## Abstract

Diabetic retinopathy (DR) is a common complication of diabetes, which can lead to vision loss. Early diagnosis is crucial to prevent the progression of DR. In recent years, deep learning approaches have shown promising results in the development of an intelligent and efficient system for DR classification. However, one major drawback is the need for expert-annotated datasets, which are both time-consuming and costly. To address these challenges, this paper proposes a novel dynamic graph clustering learning (DGCL) method for unsupervised classification of DR, which innovatively deploys the Euclidean and topological features from fundus images for dynamic clustering. Firstly, a multi-structural feature fusion (MFF) module extracts features from the structure of the fundus image and captures topological relationships among multiple samples, generating a fused representation. Secondly, another consistency smoothing clustering (CSC) module combines network updates and deep clustering to ensure stability and smooth performance improvement during model convergence, optimizing the clustering process by iteratively updating the network and refining the clustering results. Lastly, dynamic memory storage is utilized to track and store important information from previous iterations, enhancing the training stability and convergence. During validation, the experimental results with public datasets demonstrated the superiority of our proposed DGCL network.

## 1. Introduction

Diabetic retinopathy (DR) is a prevalent disease that affects the retina and it is recognized as a primary cause of vision impairment in middle-aged and older populations [1]. According to a research report by the National Institute of Diabetes and Digestive and Kidney Diseases (NIDDK) in the United States (https://www.niddk.nih.gov/health-information/health-statistics/diabetes-statistics, accessed on 2 August 2023), an estimated 34.2 million individuals, accounting for 10.5 percent of the U.S. population, are affected by diabetes, with only 26.9 million cases diagnosed. Early diagnosis of DR through regular screening is crucial for preventing visual impairment. However, the need for frequent check-ups poses a burden for a large number of individuals. Therefore, the development of an intelligent DR diagnosis system can greatly facilitate this process [2].

Ophthalmologists commonly analyze diabetic retinopathy using color fundus images, which encompass various feature components, like microaneurysms, hard exudates, hemorrhages, and the fovea. Moreover, the large number of individuals requiring screening makes manual detection alone impractical for accurate diagnosis. Therefore, the development of an automated diagnostic system for diabetic retinopathy is essential. Significant progress has been made in this field, as evidenced by notable studies [3,4,5]. Traditional image identification training methods heavily rely on high-quality, manually annotated labels. However, the process of annotating massive amounts of data is time-consuming and costly and restricts observation to only the annotated classes. Consequently, the rate of category discovery is generally reduced.

In comparison, unsupervised classification, driven by novel markers and anomaly data, offers unbiased disease classification without the need for annotated data and has the potential to quickly identify new categories. In detail, unsupervised representation learning aims to acquire meaningful image features without relying on artificial labels. Among these approaches, clustering-based representation learning methods [6,7] are recognized for their significant potential in this area. However, traditional clustering methods can capture limited intra-image invariance and inter-image similarity. In contrast, graph convolutional neural networks (GCNs) can effectively leverage image similarity. In other words, while GCNs have a significant advantage in mining contextual relationships within sample relational networks, convolutional neural networks (CNNs) excel at extracting global characteristics. This observation has been supported by numerous studies [8,9,10].

**Figure 1:**
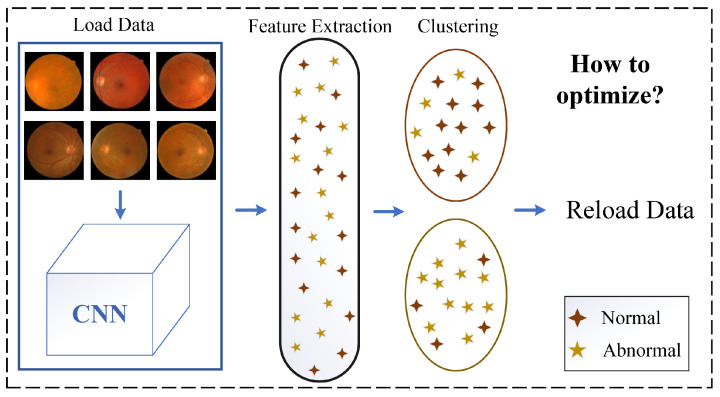
Traditional clustering-based unsupervised representation learning.

As mentioned earlier, previous unsupervised representation learning methods based on clustering differ from supervised learning in terms of their training strategies. This difference can sometimes result in reduced model stability during the training process. In Figure 1, it is evident that the network undergoes a series of operations, such as data loading, feature extraction, and feature clustering, repeatedly during model training, resulting in alternating clustering and network parameter updates. We believe that such intermittent training may result in a loss of model performance. To tackle these challenges, this study proposes a dynamic unsupervised clustering representation learning model based on a graph convolutional neural network (GCN) for classifying diabetic retinopathy. By integrating minor batch annotation updates into the model training process, this model aims to bridge the gap in training strategies between deep clustering and supervised learning, thereby enhancing the model’s robustness.

**Figure 2:**
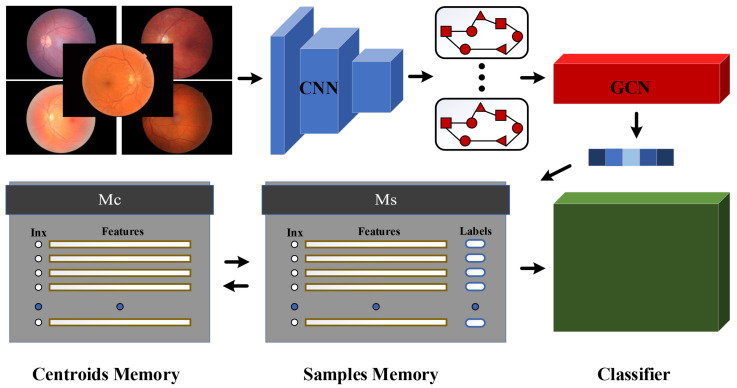
The proposed architecture of the DGCL model. Firstly, the MFF module utilizes CNN and GCN networks to extract features at the Euclidean and topological levels, respectively. This mechanism not only captures global features from the fundus image but also identifies topological associations between positive and negative samples. Subsequently, the CSC module performs deep clustering using the extracted representations, which contain rich spatial information. Importantly, the network update and deep clustering in the DGCL model occur simultaneously rather than alternately. Finally, the integration of two dynamic memory stores, $M_{s}$ and $M_{c}$, provides dynamic online support for the DGCL model, enabling continuous training without the time-consuming process of repeatedly extracting data samples.

This work presents the dynamic graph clustering learning (DGCL) model for unsupervised classification of diabetic retinopathy. The DGCL model incorporates three key modules: the multi-structural feature fusion (MFF) module, the consistency smoothing clustering (CSC) module, and an evolution strategy for pseudo-labels and clustering centroids. To enable annotation updating and centroid evolution, we propose and maintain two active memory stores denoted as $M_{s}$ and $M_{c}$. As illustrated in Figure 2, image features, along with their corresponding markers, are stored in $M_{s}$, while centroids are updated using $M_{c}$. This allows the DGCL model to be trained consecutively, mimicking the process of supervised learning. Moreover, the DGCL model eliminates the need for artificial labels during model training, leading to substantial time and cost savings. During the training process, the networks, $M_{s}$, and $M_{c}$ are updated concurrently instead of alternatively. This uninterrupted and timely evolution of the networks and memory stores ensures that the classifiers after the MFF module are trained more steadily. Consequently, the training loss of the DGCL model exhibits greater stability.

In summary, we highlight our contributions as follows:

(1) For unsupervised feature representation learning in diabetic retinopathy (DR) classification, this paper proposes a multi-structural feature fusion module that incorporates rich spatial information. Specifically, the topological and Euclidean features from different network structures are integrated into a unified representation. In comparison to traditional single-structure networks, the proposed method demonstrates enhanced feature learning capabilities in unsupervised clustering;
(2) To expedite training effectiveness, we introduce a novel consistent smoothing clustering (CSC) module that concurrently combines deep clustering and network updating. This concurrent step ensures consistent and seamless performance improvement in unsupervised DR classification;
(3) Additionally, our model incorporates a dynamic clustering memory updating mechanism. This mechanism enables simultaneous online fundus image clustering and network updating, rather than alternating between the two. As a result, the model’s robustness is improved, facilitating a smoother training process.

Overall, this paper presents an early attempt at medical image processing using multi-structural feature learning and dynamic clustering. It holds potential application value in clinical DR classification scenarios where expert labels are unavailable. Furthermore, our proposed approach, called DGCL, demonstrates broad applicability and generalization capabilities.

## 2. Related Work

This section is initiated with a review of current retinal image classification methods based on supervised learning, followed by an exploration of the applications and potential benefits of unsupervised learning methods in the medical image analysis field.

### 2.1. Deep Learning in Diabetic Retinopathy Classification

In recent years, several supervised representation learning algorithms have been developed for retinal classification [5,11,12]. For instance, Beevi et al. [5] proposed a novel multi-level severity classification for diabetic retinopathy (DR) by utilizing two-stage SqueezeNet and deep convolutional neural networks. Chen et al. [11] developed a fully automated method for assessing image quality and grading retinopathy that integrated data augmentation and employed the convolutional neural network approach with the EfficientNet-B2. Abdelmaksoud et al. [12] devised a hybrid deep learning technique named E-DenseNet that leveraged EyeNet and DenseNet models through transfer learning to detect different retinopathy grades.

Additionally, several other studies have incorporated deep learning into various medical image processing tasks [13,14,15]. Chaki et al. [13] proposed a comprehensive four-fold deep learning network for brain tumor segmentation and classification. This network encompasses the segmentation of brain tumor regions, ROI selection, feature extraction, and subsequent classification. In another notable work, Foersch et al. [14] developed and evaluated a multi-stain deep learning model to determine the Immunoscore in a cohort of over 1000 patients with colorectal cancer. Their study demonstrated the remarkable prognostic capabilities of this model, surpassing the performance of other clinical molecular and immune cell-based parameters. Furthermore, Chaki et al. [15] meticulously documented and reviewed detection and classification techniques for neurodegenerative disorders. They summarized the key findings of previous studies and underscored the significance of their conclusions.

It is worth emphasizing that the majority of existing algorithms for medical image processing heavily depend on supervised learning, which requires a significant quantity of labeled medical images. However, manual data labeling is not only costly but also demands considerable labor resources, rendering it financially impractical for many researchers. Consequently, the exploration and development of unsupervised medical image analysis techniques hold great importance and value for the advancement of AI-based medical imaging in the future. Robust unsupervised learning models have the potential to alleviate the burden of manual annotation and enable more efficient and cost-effective analysis of medical images.

### 2.2. Unsupervised Representation Learning

Recently, the potential of unsupervised learning in medical image analysis has become increasingly evident [16,17,18,19]. Among the various approaches, clustering-based representation learning techniques have emerged as a promising direction [17,18,20]. For instance, Wang et al. [17] proposed a clustering-guided contrastive learning framework for robust and accurate whole-slide image retrieval. Their method utilizes a large-scale dataset of unlabeled histopathological images. In a similar vein, Wang et al. [18] developed a multi-site clustering and nested feature extraction method for fMRI-based autism spectrum disorder, taking into account inter-site heterogeneity within each category. Furthermore, Nguyen et al. [20] addressed unsupervised learning with 2D and 3D medical data modalities by constructing a self-supervised image-volume representation learning framework based on intra-inter contrastive clustering.

However, the deep clustering methods mentioned above separate network updates and cluster evolution into alternate steps. Additionally, these methods focus solely on pixel-level features derived from the image, leaving potential for further improvement based on the consideration of their topological relations. In contrast, our proposed MFF module has the potential for extracting integrated features both at the pixel level and sample level. Furthermore, the proposed CSC module promotes simultaneous network updates and cluster evolution, thereby enhancing the overall performance of the clustering-based representation learning.

## 3. Our Proposed Dynamic Graph Clustering Learning

This section presents a detailed description of the model construction for our dynamic graph clustering learning (DGCL) approach. It also includes an in-depth discussion on the training mechanism and highlights the differences between DGCL and conventional deep clustering methods. In the DGCL approach, we leverage the multi-structural feature fusion (MFF) module to analyze topological features at the graph level and extract CNN features at the Euclidean level from fundus images. By combining these features into a multi-structural representation, we achieve a more comprehensive depiction of the image. Furthermore, the consistent smoothing clustering (CSC) method plays a crucial role in the DGCL framework. It incorporates a dynamic clustering memory updating mechanism to cluster the fused multi-structural features. This mechanism ensures consistent and seamless performance improvement throughout the model convergence process. Figure 2 provides a visual representation of the proposed method.

### 3.1. Multi-Structural Feature Fusion

For unsupervised diabetic retinopathy classification, we define the input fundus images as $X=\left\{x_{1},x_{2},\ldots ,x_{i},\ldots ,x_{N}\right\}$. To represent each retinal image, we employ two different network architectures: convolutional neural networks (CNNs) $f_{c}\left(W_{c}\right)$ and graph convolutional neural networks (GCNs) $f_{g}\left(W_{g}\right)$, which generate Euclidean and topological feature representations, respectively. The primary objective of this section is to focus on the integration of multi-structural features to achieve effective feature fusion. By merging these features, we aim to enhance the discriminative information contained within the fused representation. After constructing the network, our training objective is to evolve the network parameters $W_{c}$ and $W_{g}$ to create highly discriminative features. To facilitate this process, the initial iteration of DGCL utilizes dynamic memory stores $M_{s}$ and $M_{c}$, which are initialized using the K-means algorithm. This mechanism ensures the continuous and stable operation of the proposed model.

In the process of multi-structural feature fusion, the CNN feature extractor initially maps each fundus image to generate compact Euclidean-level features, denoted as $h^{c}=f_{c}\left(W_{c},x_{i}\right)$, and the whole dataset forms a CNN feature set $H^{c}=\left\{h_{1}^{c},h_{2}^{c},\ldots ,h_{i}^{c},\ldots ,h_{N}^{c}\right\}$. In addition, the CNN network $f_{c}\left(W_{c}\right)$ is optimized using the following objective function. It evolves in conjunction with the network parameters $W_{c}$, as depicted by Equation (1):

(1) $$L_{uc}=\min_{W_{c}}-\sum_{i=1}^{B}y_{i}\log f_{c}\left(W_{c},x_{i}\right)$$

where $y_{i}$ represents the pseudo-labels obtained from $M_{s}$, and *B* denotes the number of samples in each batch. Thus, the Euclidean-level feature representations can be extracted guided by the pseudo-labels from the K-means algorithm.

As for the topological-level feature learning, the k-nearest neighbor (KNN) algorithm is employed to construct a topology graph based on the compact representation vectors $h^{c}$. This enables us to explore the topological correlation between samples using the CNN features. To elaborate, we predefine a parameter *k* that determines the adjacency matrix in the K-nearest neighbor (KNN) algorithm. Each row of the matrix is denoted as $A_{j}^{k}=\left\{a_{j1}^{k},a_{j2}^{k},\ldots ,a_{jl}^{k},\ldots ,a_{jn}^{k}\right\}$. The value $a_{jl}^{k}$ (where *j* and *l* are both in the range from 0 to n) in the matrix $A^{k}$ indicates whether the *j*-th feature and the *l*-th feature are connected in the graph, with *n* representing the total number of CNN features. In order to determine each element of the KNN adjacency matrix, we calculate the feature similarity $d_{j}^{l}$ between $h_{i}^{c}$ and $h_{j}^{c}$. This results in a similarity matrix denoted as *D*, where the *j*-th row is defined as $d_{j}=\left\{d_{j1},\ldots ,d_{jl},\ldots ,d_{jn}\right\}$:

(2) $$d_{jl}=\sqrt{{∥h_{i}^{c}-h_{i}^{c}∥}^{2}}$$

For the *j*-th sample, we sort the similarity matrix $d_{j}$ and obtain its sorted matrix $d_{j}^{{}^{\prime }}$, where $Idx=\left\{j_{1},\ldots ,j_{m},\ldots ,j_{n}\right\}$ represents the indexes of the samples, with $j_{m}$ denoting the *m*-th closest neighbor samples to the *j*-th image. Mathematically, we define the adjacency matrix $A^{k}$ as follows:  

(3) $$A_{jl}^{k}=\left\{\begin{array}{c} 1,j_{m}\leq k\\ 0,j_{m}>k \end{array}\right.$$

Next, after obtaining the CNN feature collection $H^{c}=\left\{h_{1}^{c},h_{2}^{c},\ldots ,h_{i}^{c},\ldots ,h_{N}^{c}\right\}$ and its adjacency matrix *A*, we employ a graph convolutional neural network (GCN) to extract graph representations that are more abstract than the CNN ones; e.g., $h^{g}=f_{g}\left(W_{g},h_{i}^{c},A\right)$ and $H^{g}=\left\{h_{1}^{g},h_{2}^{g},\ldots ,h_{i}^{g},\ldots ,h_{N}^{g}\right\}$.

After obtaining the Euclidean and topological representations for each fundus image, we combine them into a unified feature to leverage their specific characteristics for diabetic retinopathy classification. The fusion of the final multi-structural features for each fundus image is performed as follows:

(4) $$h_{i}=\mathrm{concat}\left[h_{i}^{c},h_{i}^{g}\right]$$

Therefore, the fused multi-structure feature set is $H=\left\{h_{1},h_{2},\ldots ,h_{i},\ldots ,h_{N}\right\}$. Assisted by the corresponding annotations $Y=\left\{y_{1},y_{2},\ldots ,y_{i},\ldots ,y_{N}\right\}$, the model reads from the sample store $M_{s}$, as shown in the following formula, and $f_{g}\left(W_{g}\right)$ evolves along with the network parameters $W_{g}$:

(5) $$L_{ug}=\min_{W_{g}}-\sum_{i=1}^{B}y_{i}\log P\left(W_{g},H\right)$$

where P(…) represents the prediction layer after the feature fusion layer and $y_{i}$ denotes the pseudo-label from $M_{s}$.

Through the training process described in Equation (5), the classification ability of DR is further enhanced by leveraging dynamic clustering within the framework of multi-structural feature fusion. However, one major challenge that remains is how to improve the efficiency of the proposed dynamic clustering algorithm.

#### Consistency Smoothing Clustering Module

In the aforementioned multi-structural feature fusion, another major issue is the training efficiency between the feature learning and clustering steps. Here, we developed the consistency smoothing clustering (CSC) module to ensure consistent and seamless performance improvement during model training.

Specifically, we employ the L2 paradigm to normalize $H^{g}$ and update the sample store $M_{s}$ as follows:

(6) $$H_{\lambda }\leftarrow \lambda \frac{H}{{∥H∥}_{2}}+\left(1-\lambda \right)H_{\lambda }$$

where $H_{\lambda }$ represents the representation in $M_{s}$, and λ∈(0,1] denotes the momentum coefficient. Simultaneously, we update the pseudo-label for each representation in $M_{s}$ based on its closest center of mass using the following formula:

(7) $$L_{uce}=\min_{y^{c}\in \left\{1,\ldots ,C\right\}}{∥h-C_{y^{c}}∥}_{2}^{2}$$

where $C_{y^{c}}$ is the centroid representation of category $y^{c}$.

Finally, we update all centroids in $M_{c}$ every 10 iterations by calculating the average of the representations belonging to their respective centroid. This ensures that the centroids accurately reflect the aggregated information for the associated samples.

In summary, the proposed dynamic graph clustering learning (DGCL) model is optimized by minimizing the following objective function:

(8) $$\min L=\lambda \left(L_{uc}+L_{ug}\right)+\left(1-\lambda \right)L_{uce}$$

In this way, the DGCL model is trained in a manner akin to supervised learning. The complete algorithm is summarized in Algorithm 1.
**Algorithm 1** DGCL**Input:**$\;X=\left\{x_{1},x_{2},\ldots ,x_{i},\ldots ,x_{N}\right\}$**initial:**$\;g_{0}=0$, $d_{0}=0$, K-means for $M_{s}$**Repeat:**       Send the images x∈X to the CNN and map images into compact feature vectors $h^{c}=f_{c}\left(W_{c},x_{i}\right)$;       Update the CNN via Equation (1);       Calculate the similarity between images via Equation (2) and formulate the adjacent matrix $A^{k}$ to construct the relation graph *G* via Equations (2) and (3);       Send $H^{c}=\left\{h_{1}^{c},h_{2}^{c},\ldots ,h_{i}^{c},\ldots ,h_{N}^{c}\right\}$ and its adjacency matrix *A* to the GCN module and output the graph representations $H^{g}=\left\{h_{1}^{g},h_{2}^{g},\ldots ,h_{i}^{g},\ldots ,h_{N}^{g}\right\}$;       Obtain pseudo-labels $Y=\left\{y_{1},y_{2},\ldots ,y_{i},\ldots ,y_{N}\right\}$ for this batch from the sample memory $M_{s}$;       Update the GCN module with stochastic gradient descent via Equation (5);       Read pseudo-labels $Y=\left\{y_{1},y_{2},\ldots ,y_{i},\ldots ,y_{N}\right\}$ for this batch from the sample memory $M_{s}$;       Update sample memory via Equation (6);       Update centroid memory every 10th iteration via Equation (7);**Until** Convergence;**Prediction** The category of retinal images.

### 3.2. Dealing with Clustering Distribution

**Weight Allocation.** The deep clustering (DC) approach commonly utilizes uniform sampling in each iteration to avoid the formation of excessively large clusters during the training process. In contrast, the dynamic graph convolutional clustering (DGCL) method enables dynamic changes in cluster sizes during each iteration. Uniform sampling necessitates resampling of the entire training data in each iteration, which is redundant and computationally expensive. To overcome this issue, we propose a novel strategy: adjusting weight allocation based on the cluster sizes within each category. To validate the effectiveness of this approach, we incorporate the weight allocation into a DC model. Through empirical analysis, we observed that the model’s performance remained stable when the weight was determined as $W_{y^{c}}\propto \frac{1}{\sqrt{N_{c}}}$, where $N_{y^{c}}$ represents the number of features in category $y^{c}$ and $W_{y^{c}}$ denotes the corresponding weight. As a result, we apply the same weight allocation method to DGCL. This adjustment allows each cluster’s representations to contribute more significantly to the network updating process, effectively pushing the decision boundary outward and capturing a greater number of potential features.

**Feature Dimensionality Reduction.** Conventional networks like AlexNet and ResNet-50 typically generate high-dimensional image representations, such as 4096 or 2048 dimensions. However, this leads to high computational resource requirements for subsequent clustering tasks. DC methods often utilize principal component analysis (PCA) to reduce the dimensionality of features across the entire dataset. However, this approach is not feasible for DGCL due to the computational cost incurred by conducting PCA in each iteration.. To address this issue, we deploy a nonlinear layer—specifically, fc-bn-reludropout-fc-relu—to reduce the dimensionality of fundus image representations to 256 dimensions. During the DGCL training phase, this nonlinear layer is jointly optimized but subsequently frozen for downstream tasks.

### 3.3. Differences from Deep Clustering

In deep clustering (DC), the process of identifying salient features entails alternating between updating the network with pseudo-labels and intermittently performing representation clustering. To extract deep features using a global clustering algorithm, such as the K-means algorithm, the DC mechanism leverages the complete training dataset. Moreover, these clustering approaches entail extensive shuffling of pseudo-labels, necessitating the rapid adaptation of the networks to varying annotations in subsequent iterations. This iterative adjustment helps the network learn more robust and discriminative features. Through iterative refinement of the network and clustering results, DC aims to enhance the distinction between different clusters in the feature space, thereby improving the clustering performance.

The proposed DGCL method deviates from DC in several aspects. Firstly, DGCL concentrates on learning both intra-image invariance and inter-image similarities in retinal images. This allows DGCL to capture both local and global image characteristics, enhancing the representation learning process. Secondly, DGCL eliminates the need for re-extraction of representations. Instead, it leverages two memory stores; namely, $M_{s}$ and $M_{c}$. $M_{c}$ stores category centroids, which are the mean features of all images in a particular category. On the other hand, $M_{s}$ stores representations and fake labels for the entire dataset. These memory stores facilitate the steady evolution of pseudo-labels alongside the network parameters. The categories in DGCL are not fixed but are updated continuously during training, allowing the network and pseudo-labels to evolve in parallel. Through continuous iterations of the proposed DGCL model, both the network and pseudo-labels evolve simultaneously, leading to enhanced clustering performance. In summary, DGCL combines intra-image invariance learning, similarity learning, and memory stores to achieve effective representation learning and clustering in a unified framework. Overall, our DGCL approach offers a novel perspective on dynamic graph clustering learning, providing valuable insights into the integration of multi-structural features for enhanced medical image analysis.

## 4. Experiments

### 4.1. Database Description

This paper utilizes two widely recognized public fundus image databases for conducting experiments. The distribution of classes within the datasets is presented in Table 1. Clinicians assessed the severity of diabetic retinopathy for each sample in the databases, using a categorical scale ranging from 0 to 4. Each category corresponds to the following scale: no DR, mild DR, moderate DR, severe DR, and proliferative DR. Specifically, category 0 is categorized as normal DR, while categories 1, 2, 3, and 4 are grouped as abnormal DR.

For our first database, we utilized the Kaggle dataset, comprising 88,400 retinal images acquired under diverse imaging conditions. The collection of retinal images was graciously provided by EyePACS [21], a public platform for diabetic retinopathy diagnosis. The second database employed in this study was the Messidor-2 dataset [22], encompassing diabetic retinopathy examinations contributed by the Messidor program partners and Brest University Hospital. Each examination in this database comprises a pair of two retinal images with the macular as the central focus. In total, Messidor-2 comprises 1748 images.

### 4.2. Implementation Details

**Pretraining and Preprocessing.** Resnet-50 was chosen as the backbone for our convolutional neural network (CNN), and we constructed a graph using the k-nearest neighbor (KNN) approach for the graph convolutional neural network (GCN) module, which consisted of two graph convolution layers. Before training, we initialized the CNN using the pretrained Resnet-50 model from ImageNet. Furthermore, we preprocessed the retinal images by cropping them, eliminating black regions, and focusing the neural network’s attention on the pertinent areas for recognition. Next, we resized the retinal images to a resolution of 224 × 224 pixels, constrained by the graphics memory. For enhanced training, we applied data augmentation techniques to the retinal images, including horizontal flipping, random rotation, and vertical flipping. Color jittering was implemented using PyTorch 1.8, with the following ranges for factors: saturation (0 to 2), contrast (0.5 to 1.2), hue (−0.6 to 0.6), and brightness (0.5 to 1.2). To introduce color variation and discourage the model from overemphasizing irrelevant color details, color jittering was randomly applied to the training images.

**Experimental parameters of DGCL.** We devised a dynamic learning rate adjustment strategy. Initially, the learning rate is set to a constant value of 0.01 for the initial 30 epochs. Subsequently, it gradually decreases as the training proceeds. We set the batch size to 128, the number of clusters to 5, the momentum factor (*m*) to 0.5, and the balance factor (λ) to 0.6. We distributed all the data across two GPUs for processing. Based on our evaluation of the model’s efficiency and performance, we decided to update the centroid memory ($M_{c}$) every 10 epochs. Drawing from our prior experience in constructing GCN graphs, we empirically set the parameter *k* to 4.

### 4.3. Evaluation and Result

Utilizing the Kaggle DR detection and Messidor-2 retinal datasets, we assessed the classification performance of our proposed DGCL method based on metrics such as accuracy (ACC), specificity (SPE), and sensitivity (SEN). Based on these measurements, we conducted the following experiments: (1) Initially, we trained the DGCL model in its original form to evaluate the contributions of the MFF and CSC modules. (2) Next, we generated ROC curves and t-SNE plots utilizing the experimental results obtained in the proceeding step. (3) Lastly, we conducted a comparative analysis of DGCL and other existing approaches to demonstrate its superiority.

#### 4.3.1. Results

The summarized experimental results for our DGCL model and the compared method are presented in Table 2. It is evident from the table that our DGCL model achieved excellent performance in classifying the retinal images across both datasets. Specifically, on the Kaggle dataset, the DGCL model achieved a sensitivity of 82.8%, specificity of 99.6%, and accuracy of 91.2%. On the Messidor-2 dataset, the DGCL model achieved a sensitivity of 87.6%, specificity of 99.8%, and accuracy of 93.7%.

**Figure 3:**
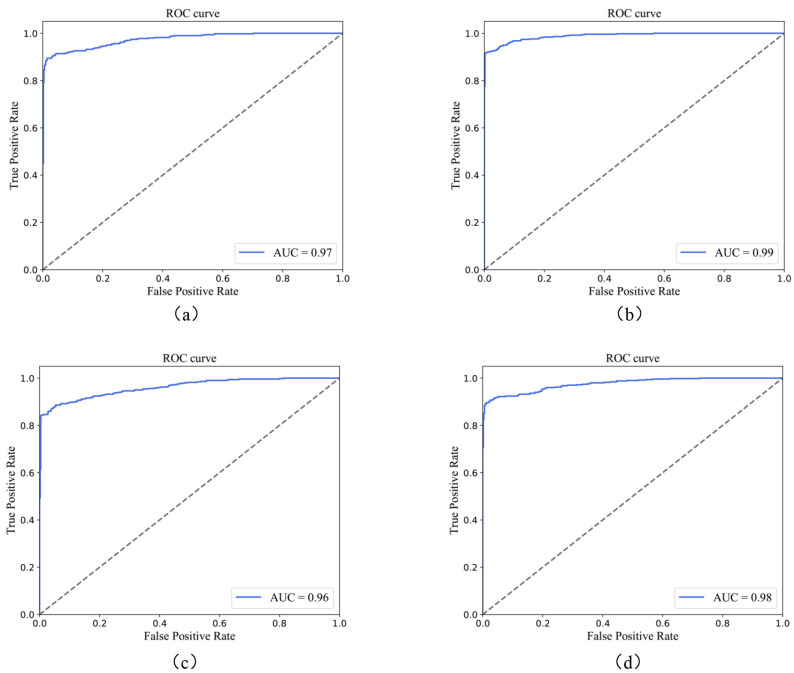
(**a**,**b**) ROC curves of the DGCL model for DR classification with the Kaggle and Messidor-2 datasets, respectively. (**c**,**d**) ROC curves of the DCCL model with the Kaggle and Messidor-2 datasets.

Figure 3 presents the receiver operating characteristic (ROC) curves of our DGCL model and its modified version DCCL (DGCL without the MFF module). As depicted in Figure 3a,b, DGCL achieved superior performance, with an AUC of 0.97 with the Kaggle database and 0.99 with the Messidor-2 database, outperforming DCCL, which had an AUC of 0.96 with the Kaggle database and 0.98 with the Messidor-2 database. These results indicate that our DGCL model can achieve excellent performance in ROC analysis, and the proposed MFF module plays a significant role in extracting the similarity relationships among retinal images, thereby contributing to the task of unsupervised diabetic retinopathy classification. Additionally, the ROC curves of the Kaggle dataset exhibited a smoother trend compared to those of the Messidor-2 dataset. The discrepancy can be attributed to the larger size of the testing set in the Kaggle database, which provides a more robust evaluation of the model’s performance.

**Figure 4:**
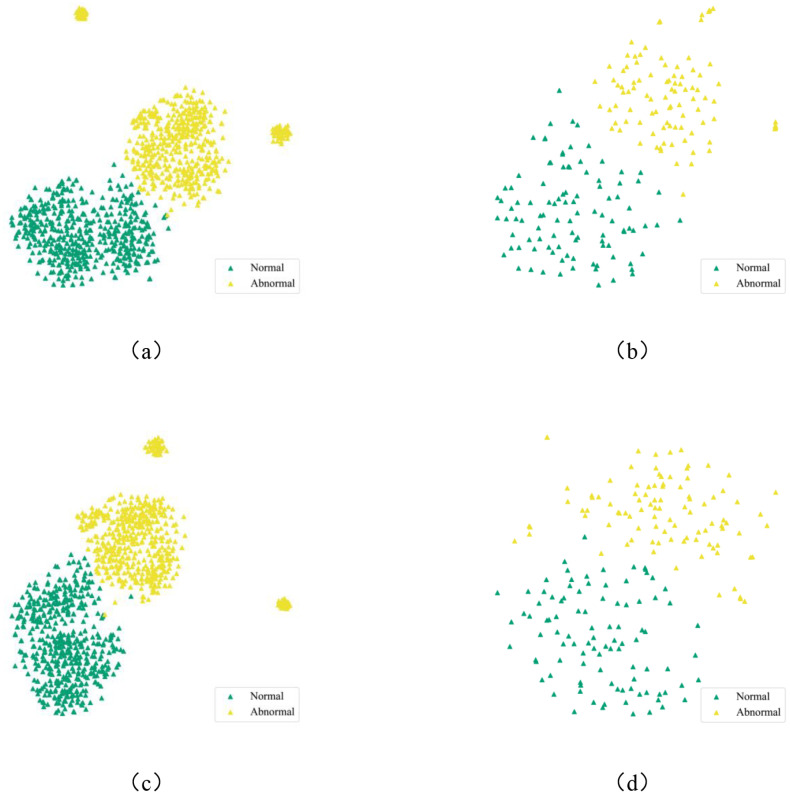
(**a**,**b**) t-SNE visualization of the DGCL model for DR classification with the Kaggle and Messidor-2 datasets, respectively. (**c**,**d**) t-SNE of the DCCL model with the Kaggle and Messidor-2 datasets.

In order to further evaluate the classification performance of DGCL, we utilized t-distributed stochastic neighbor embedding (t-SNE) to visualize the retinal features extracted by the DGCL model for each class. The visualization results are depicted in Figure 4a,b. As depicted in the figure, DGCL successfully separated the retinal images into two distinct categories. This finding suggests that the classification performance of DGCL in precisely categorizing retinal images remains robust, even in the absence of annotated data. Therefore, DGCL demonstrates the capability to accurately classify retinal categories, even in an unsupervised setting. Moreover, the utilization of t-SNE visualization provides valuable insights into the discriminative power of the extracted retinal features, highlighting the effectiveness and efficiency of the DGCL model in capturing the underlying patterns and characteristics of the retinal images.

#### 4.3.2. Comparison with State-of-the-Art Models

From Table 2, it is evident that the classification performance with the Kaggle database surpassed that with the Messidor-2 database. This difference in performance may be attributed to the discrepancies in image quantity and quality between the two databases. For the binary classification of diabetic retinopathy, our DGCL model was compared with baseline methods, as presented in Table 2. The results in this table reveal that DGCL achieved superior performance in both databases, highlighting the efficacy of the MFF module in facilitating robust training of DGCL. By employing appropriate training strategies, DGCL achieved an accuracy of 91.2% with the Kaggle dataset and 93.7% with the Messidor-2 dataset. Notably, DGCL outperformed other methods in terms of accuracy with both datasets. Similarly, DGCL demonstrated favorable sensitivity results, achieving 82.8% with the Kaggle dataset and 87.6% with the Messidor-2 dataset. Furthermore, DGCL attained the highest specificity performance compared to other methods. Overall, DGCL exhibited high accuracy and low rates of missed and misdiagnosed cases. These findings underscore the potential of our proposed method for clinical diagnosis of diabetic retinopathy.

### 4.4. Further Analysis

In this section, we evaluate the performance of the proposed DGCL model under various conditions and provide a summary of the obtained results.

#### 4.4.1. DGCL without MFF Module

Initially, we examined the impact of dynamic clustering on the performance of image classification by isolating the MFF module from the DGCL model. The performance of the DGCL model without the MFF module, referred to as DCCL, is presented in Table 2. The results indicate that DCCL achieved accuracy of 89.8%, sensitivity of 80%, and specificity of 99.6% with the Kaggle database. With the Messidor-2 database, DCCL achieved accuracy of 92.6%, sensitivity of 85.8%, and specificity of 99.4%. These findings emphasize the effectiveness of dynamic clustering in improving the performance of DR classification.

Furthermore, we utilized ResNet-50 as the backbone network and drew inspiration from two widely used frameworks in the semi-supervised learning field; namely, FixMatch [29] and MixMatch [28]. To assess the performance of DGCL with limited labeled data, we conducted experiments using only 1% of the labeled data from the Kaggle dataset, and the results are summarized in Table 2. The results demonstrate that the CSC module enhances model stability, building upon traditional deep clustering methods and further boosting overall performance.

We also employed statistical analysis to evaluate the performance of the proposed DGCL model without the MFF module, utilizing the area under curve (AUC) and receiver operating characteristic (ROC) curves. These results are visualized in Figure 3c,d. As depicted in these diagrams, the DCCL model achieved an impressive AUC of 0.96 with the Kaggle database and 0.98 with the Messidor-2 database. These findings indicate that, even in scenarios where the MFF module is not available, DGCL maintains favorable performance. This observation validates the correctness of the assumption underlying the CSC module, where an alternating process of cluster evolution and network updates does not compromise the overall effectiveness of the model. Moreover, the t-SNE performance of DGCL and DCCL (Figure 4) suggests a similar conclusion.

#### 4.4.2. Influence of Different Clustering Algorithm

This paper presents a dynamic deep clustering framework that integrates deep learning and clustering algorithms in a more efficient manner. The choice of clustering algorithm plays a crucial role in constructing the storage memory $M_{c}$. Therefore, we evaluate the impact of different clustering algorithms.

As shown in Table 2, we replaced the original K-means algorithm with the fuzzy C-means (FCM) clustering algorithm. It achieved accuracies of 0.905 and 0.932 with the Kaggle and Messidor-2 datasets, respectively. Other metrics also demonstrated satisfactory performance comparable to the K-means algorithm. These results indicate that our proposed dynamic graph clustering mechanism can be applied with different clustering algorithms, effectively improving training efficiency through our novel frameworks.

#### 4.4.3. Influence of the Centroid Frequency

**Figure 5:**
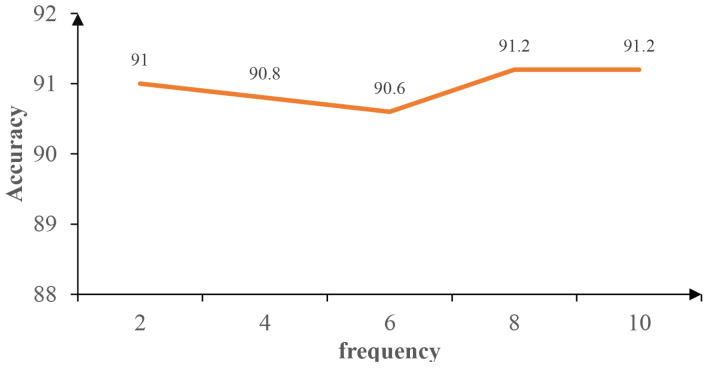
Accuracy of DGCL with the Kaggle dataset for different centroid update frequencies.

The DGCL model incorporates a hyperparameter that determines the frequency of evolution of the centroid storage. To examine the impact of this specific hyperparameter, we performed experiments with a subset of the Kaggle dataset and evaluated the classification accuracy of the DGCL model. Figure 5 illustrates the relationship between the evolutionary frequency of the centroid store and the performance of the DGCL model. Based on our analysis, we observed that the model’s performance was not significantly affected as the frequency decreased. This suggests that our approach is not highly sensitive to changes in the frequency parameter, as long as it is properly adjusted. Therefore, we employed center-of-mass memory updates every 10 epochs as our chosen frequency for the DGCL model.

#### 4.4.4. Influence of the Backbone

**Figure 6:**
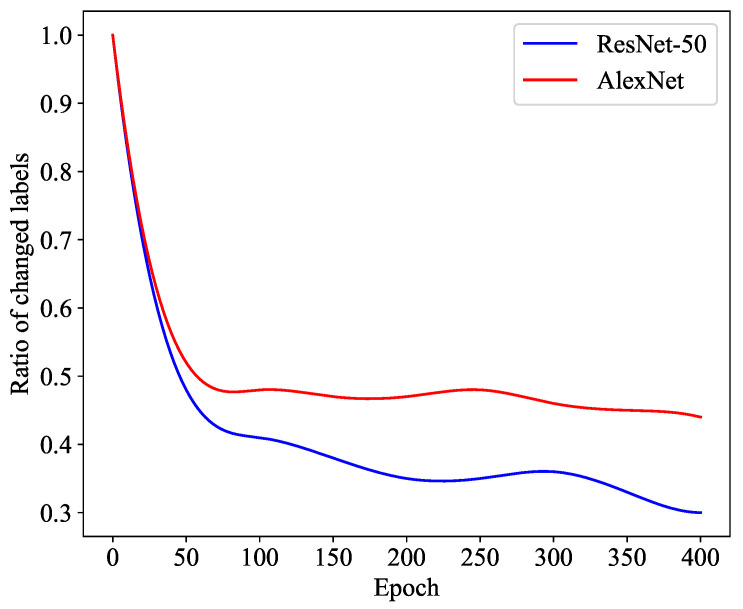
The proportion of samples reassigned to new pseudo-labels in each epoch during training.

Figure 6 demonstrates the stability and convergence of the DGCL model throughout the evolving iterations. To evaluate the stability of our proposed DGCL, we monitored the number of features whose pseudo-labels changed per batch and calculated the corresponding ratio. A lower number of annotation changes indicates improved robustness. We present the results for two network backbones, ResNet-50 and AlexNet, which were trained from scratch using the DGCL approach. As shown in the curves, during the initial training period, almost all samples underwent reassignment of pseudo-labels in each epoch. However, as the networks were updated, the proportion of samples with pseudo-labels requiring reassignment decreased significantly. After 50 epochs, the changing ratio gradually decreased and stabilized within a low range. Although a few features continuously adjusted their annotations, the DGCL model eventually reached a stable state. As observed in Figure 6, ResNet-50 exhibited greater stability compared to AlexNet. Considering this observation, we selected ResNet-50 as the preferred backbone for the current version of DGCL.

#### 4.4.5. Influence of the Balance Coefficient λ

**Figure 7:**
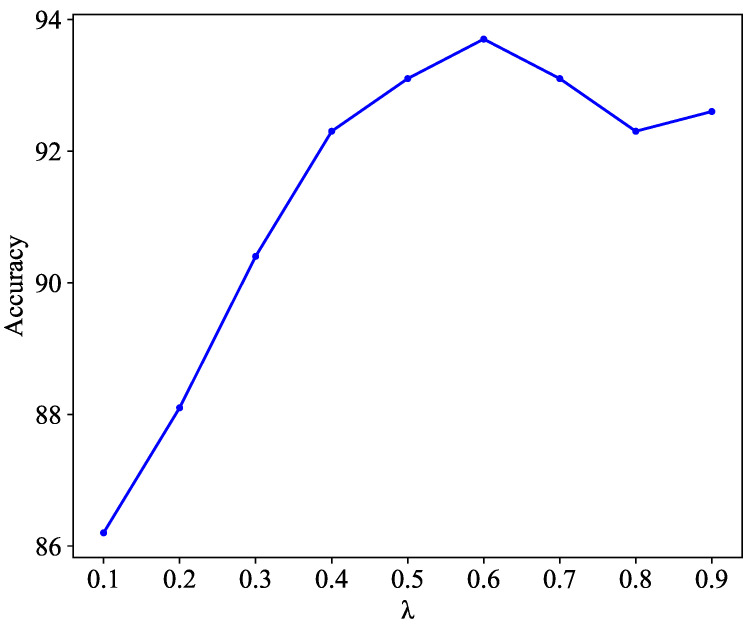
Variation in the performance of DGCL with different balance coefficients λ with Messidor-2.

The training objective in Equation (8) includes a parameter λ that balances the weight between the multi-structural feature fusion and consistency smoothing clustering modules. To evaluate their importance, we varied the value of λ from 0.1 to 0.9, and the performance results are depicted in Figure 7. Notably, the model achieved optimal performance when λ was set to 0.6. This indicates that slightly more weight was assigned to network updates compared to the evolution of centroids.

#### 4.4.6. The Resource Analysis of DGCL

**Figure 8:**
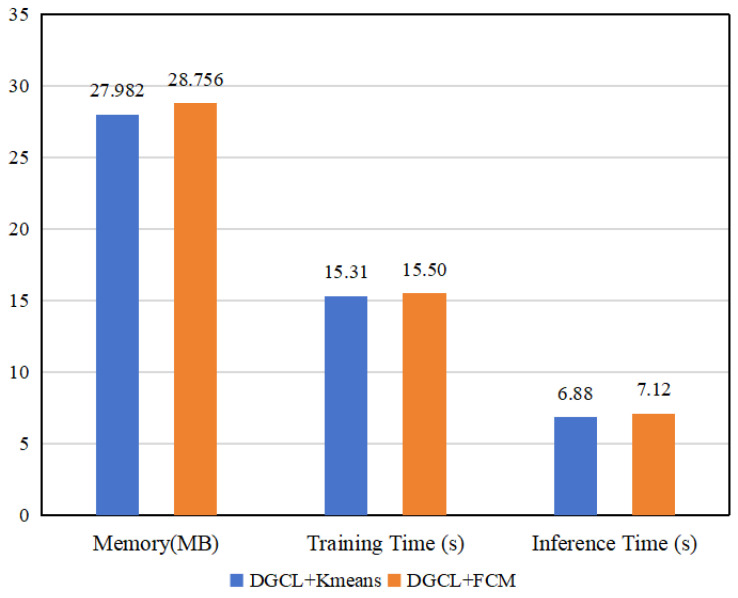
Comparative analysis of computing resource occupation with Messidor-2.

The proposed dynamic clustering mechanism enhances the training process involving deep learning representation and clustering updating. To show its superiority, we calculated the memory footprint, training time, and inference time of DGCL with different clustering algorithms, as depicted in Figure 8. We specifically focused on the memory footprints during the clustering step, model training, and inference for each image using different clustering algorithms (K-means and FCM) with the Messidor-2 dataset.

The results demonstrate that DGCL only requires a memory footprint of approximately 27–28 MB per clustering step. Moreover, the average training time was around 15 ms, while the inference time ranged from 6 to 7 ms for processing each image. These findings indicate that our approach maintains a relatively low memory footprint regardless of the chosen clustering algorithm. This emphasizes the efficiency and scalability of our method, making it suitable for deployment in resource-constrained clinical DR diagnosis scenarios.

## 5. Discussion and Conclusions

In this paper, we propose a novel approach named dynamic graph clustering learning (DGCL) for diabetic retinopathy (DR) classification. Our method incorporates two key modules: the multi-structural feature fusion (MFF) and the consistency smoothing clustering (CSC) modules. Firstly, we introduced the MFF module, which allows us to explore relationships from unlabeled data and improve early self-supervised representation learning techniques. This module has the potential to improve the performance of our model by leveraging valuable information from unlabeled data. Next, we discussed the CSC module, which removes the need for manual annotation and enables our model to be trained efficiently, comparable to supervised learning methods. The CSC module plays a vital role in achieving accurate DR classification without relying on artificial labels. Moreover, we defined two memory stores that enable uninterrupted dynamic clustering in DGCL, enhancing the model’s robustness and improving training strategies. The experimental results with both the Kaggle and Messidor-2 datasets demonstrate the superiority of our proposed DGCL method compared to several baseline methods. This approach shows promising potential for diabetic retinopathy recognition in fundus images.

The DGCL method achieves impressive results without using manual labels, which represents a valuable exploration in the field of unsupervised learning. As the volume of medical image data continues to grow, relying solely on manual labeling is becoming inefficient and resource-intensive. Therefore, unsupervised methods hold great promise for future applications.

However, it is important to note that DGCL currently only partially surpasses supervised methods and is not yet comparable to some advanced supervised methods in terms of achieving maximum performance. Supervised methods trained on a large number of labeled samples are only limited by computational resources, albeit with a high cost in terms of economics and time. Therefore, unsupervised DR classification offers an innovative avenue for medical image classification. In future work, we will focus on refining DGCL’s fine-grained features to further improve the performance of unsupervised frameworks for binary and even multi-class classification tasks.

## Figures and Tables

**Table 1:** The numbers of images in the experimental datasets.

Label	Kaggle	Messidor-2
No DR	65,343	1805
Mild DR	6205	370
Moderate DR	13,153	999
Severe DR	2087	193
Proliferative DR	1914	295

**Table 2:** Experimental results of DGCL and compared methods.

Methods	Kaggle			
	**Accuracy**	**Sensitivity**	**Specificity**	**AUC**
Hagos et al. [23]	0.909	-	-	-
Garcia et al. [24]	0.837	0.545	0.937	-
Raja et al. [25]	0.874	0.769	0.936	-
Yu et al. [26]	0.855	0.937	-	-
Zhang et al. [27]	0.899	0.882	0.913	-
MixMatch [28]	0.821	0.678	0.964	0.87
FixMatch [29]	0.835	0.692	0.978	0.9
DCCL	0.898	0.8	0.996	0.96
DGCL+FCM	0.905	0.875	0.916	0.95
DGCL	0.912	0.828	0.996	0.97
	**Messidor-2**			
	**Accuracy**	**Sensitivity**	**Specificity**	**AUC**
De et al. [30]	0.91	0.911	0.908	-
Zhang et al. [27]	0.918	0.902	0.93	-
MixMatch [28]	0.866	0.738	0.994	0.94
FixMatch [29]	0.888	0.778	0.998	0.97
DCCL	0.926	0.858	0.994	0.98
DGCL+FCM	0.932	0.879	0.99	0.97
DGCL	0.937	0.876	0.998	0.99

## Data Availability

Data sharing not applicable.
